# Psychometric Properties of the Mobile Health Literacy Scale in the Workers of an Automotive Metal Sheet Factory in Shahrekord, Iran

**DOI:** 10.3928/24748307-20220921-01

**Published:** 2022-10

**Authors:** Parastoo Yarmohammadi, Mohammad Ali Morowatisharifabad, Sayyed Saeid Khayyatzadeh, Farzan Madadizadeh, Zohreh Rahaei

## Abstract

**Background:**

Mobile health (mHealth) literacy refers to the ability to use mobile devices to search, find, understand, evaluate, and use health information to identify or solve a health problem. Health literacy skills are important for improving health information interventions and it will not be possible to investigate this skill unless a valid and reliable tool is developed.

**Objective:**

This study aimed to investigate the psychometric properties of the Persian version of the mHealth Literacy Scale in the workers of an automotive metal sheet factory in Shahrekord, Iran.

**Methods:**

After forward and backward translation of the scale and ensuring the accuracy of the translation, qualitative face validity was examined by an expert panel and quantitative face validity by 10 workers using the item impact score. Content validity index (CVI) and content validity ratio were investigated by seven experts on health education. To investigate construct validity, the scale was completed by 200 workers. One-factor and two-factor structures of the measure were studied using confirmatory factor analysis and the reliability was evaluated using Cronbach's alpha coefficient.

**Key Results:**

The CVI for each of the three parameter (relevance, clarity, simplicity) was rated 0.75 to 1 for each item. Confirmatory factor analysis showed that the one-factor model had a better fit to the data than the two-factor model [goodness of fit index = 0.985(>0.90), comparative fit index = 0.999 (>0.90), Tucker-Lewis index = 0.996 (>0.90), normed fit index = 0.994(>0.90), root mean square error of approximation = 0.038(< 0.08)]. Furthermore, the scale had an acceptable internal consistency (Cronbach's alpha = 0.964).

**Conclusion:**

The Persian version of mHealth Literacy Scale has satisfactory reliability and validity and could be used as an effective tool to evaluate mHealth literacy among Iranian workers. [***HLRP: Health Literacy Research and Practice*. 2022;6(4):e257–e261.**]

**Plain Language Summary:**

This cross-sectional study was conducted on 200 workers of an automotive metal sheet factory in southwest Iran to investigate the psychometric properties of the Persian version of the mHealth Literacy Scale. The results demonstrated that one-factor structure was more appropriate for evaluating mHealth literacy among Iranian workers.

Over the past decade, the use of mobile phones has expanded rapidly worldwide. By increasing the number of mobile phone users and the expansion of smartphones, there is an opportunity to improve health care delivery by using mobile health (mHealth) in low-and middle-income countries such as Iran (Roess, 2017). mHealth refers to the use of mobile devices and communication technologies (e.g., mobile phones, tablets) for the delivery of health services and health-related information. The World Health Organization (WHO) defines mHealth as the development of mobile technologies and wireless networks to improve health outcomes, health services, and health research in providing health care and resolving health priorities (Agnihothri et al.,2020; WHO, 2011). The results of a meta-analysis showed that mHealth interventions had more beneficial effects on improving health outcomes than comparators (Yang & Van Stee, 2019).

To understand and effectively use mobile health technologies, users need to enjoy a satisfactory level of mhealth literacy. The term “mHealth literacy” is applicable to people who use different health services by means of their smartphones. Thus, the concept of mHealth literacy refers to the ability to use mobile phones to search, find, understand, evaluate, and use health information to identify or solve a health problem (Lin & Bautista, 2017).

mHealth literacy plays an important role in studying people's ability to accept and understand their health care or treatment-related information for decision-making (Lin & Lou, 2021). Therefore, it is therefore to use reliable tools to measure mHealth literacy before the use of any mHealth device, regardless of its use for self-management behaviors, access health information, and communicate with healthcare providers. In 2017, the mHEALS (mHealth Literacy Scale) was developed by Lin & Bautista (2017) in a study with university students in Taiwan. The internal consistency of the 8-item scale was evaluated and found acceptable, with Cronbach's alpha ranging from 0.75 to 0.85. Increasing mHealth literacy in the workplace is aimed at empowering individuals to make appropriate health-related decisions. Worker with higher literacy skills can better understand basic health information and services (Güner & Ekmekci, 2019). Although a high proportion of the workers need health information and advice, it has been shown that many of them are unable to find it (Dryson, 1993; Rhebergen, Lenderin et al., 2012; Rhebergen, Van Dijk et al., 2012; Rollin et al., 2013). In the health literacy survey performed by Kendir et al. (2018), workers with low education and people with low socioeconomic status have higher risk factors for having low health literacy. Furthermore, mHealth literacy is an important determinant of individual health. It is necessary to use a standard tool to measure mHealth literacy in various populations to use it more widely. No study has yet been conducted to investigate the mHealth literacy of industrial workers in low- and middle-income countries. Therefore, the present study was aimed to investigate the psychometric properties of the Persian version of the mHEALS in the workers of an automotive metal sheet factory in Shahrekord, Iran.

## Methods

### Study Design and Participants

The participants of this cross-sectional study included the workers of an automotive metal sheet factory who were enrolled using convenience sampling. The data collection procedure was performed between December 2020 and January 2021 in Sharekord, Iran. The sample size for the confirmatory factor is recommended to be 5 to 20 per item (Munro, 2005; Polit, 2017). As the mHEALS has 8 items, the sample size was decided to be 200 people.

To comply with the research ethics, the data were collected after obtaining the necessary permissions from the Vice Chancellor for Research of Shahid Sadoughi University of Medical Sciences at Yazd, Iran and the ethics code (IR.SSU.SPH.REC.1399.187). Moreover, the informed consent form was completed by all the participants after the necessary explanations regarding how to complete the questionnaire and the study objectives were given to them.

In this study, the mHEALS developed by Lin and Bautista (2017) was used. The mHEALS includes 8 items and two dimensions, consisting of health information seeking by a mobile phone (4 items) and health information evaluation (4 items), rated on a five-point Likert scale (5 = *strongly agree* to 1 = *strongly disagree*) for both dimensions. To assess the psychometric properties of the scale, first, mHEALS was translated into Persian by two translators separately and a Persian version was prepared after comparing the two versions to remove potential ambiguities and reach a consensus among experts and readers of the scale. Then, backtranslation from Persian into English was done by two other English language experts who did not know the content of the original version. Afterwards, the English version was compared with the original version and after approval of the translation, the final version of the Persian duplicate was prepared.

### Face Validity

To confirm the qualitative face validity of the scale, it was presented to eight health education specialists. The levels of difficulty (difficulty understanding phrases and words), the degree of appropriateness (appropriateness of the desired relationship between phrases and the dimensions of the scale), and ambiguity (the possibility of misunderstandings of phrases or the existence of ambiguities in the meanings of words) were examined. Minor and necessary changes were made to fully clarify the cases. The quantitative face validity of the scale was confirmed after drawing the opinions of 10 workers (other than participants) regarding the importance of the items to calculate the impact score.

### Content Validity

To measure content validity, content validity ratio (CVR) was used according to Lawshe's method. To this end, eight health education experts were asked to determine the degree of necessity of each item in a three-point Likert scale [*necessary* (3), *useful but unnecessary* (2), and *unnecessary* (1)] and then with reference to Lawshe's table, the minimum acceptable values were calculated (Lawshe, 1975). Besides this, for the content validity index (CVI) of Waltz and Bausell (1981), the relevance, clarity, and simplicity of the item were on a 4-point scale (1–4). If the scores of each item on each of the parameters were over or equal to 0.79, the item would be kept (Field, 2013).

### Construct Validity

Confirmatory factor analysis (CFA) was used to evaluate factor structure. For this purpose, several fit indices were used to examine the data-model fit in the CFA including comparative fit index (CFI) >0.9, goodness of fit index (GFI) > 0.9, Tucker-Lewis index (TLI) >0.9, normed fit index (NFI) >0.9, and root mean square error of approximation (RMSEA) <0.08 (Cheng et al., 2016; Lin et al., 2020).

### Reliability

To measure the internal consistency of the scale, Cronbach's alpha coefficient was calculated for both dimensions as well as for the whole questionnaire. Cronbach's alpha coefficients of 0.7 or higher indicate acceptable reliability (Nunnally & Bernstein, 1994).

Data were analyzed using SPSS 25.0 and Amos 24.0 software (IBM). Sociodemographic information was expressed as frequency.

## Results

### Face Validity

Regarding the qualitative face validity of the scale, based on the comments received from health education experts, minor and necessary corrections were made to increase the clarity of some of the items. For quantitative face validity, after studying of the opinions of 10 workers regarding the importance of the items, the impact scores of all items were obtained higher than 1.5, and therefore all items of the Persian version of mHEALS were kept.

### Content Validity

In this study, the CVRs of all items were estimated to be 0.75 to 1 that are desirable. As well, CVI was calculated as 0.97 to 0.8 for all items, and therefore no item was deleted at this stage.

Demographic information of participants is shown in **Table 1**. The mean age of participants was 37.97 ± 6.50 years and most (87%) of them were men. Most (57%) of them had a bachelor's degree, were married (85%), and had middle-income economic status (49.5%).

**Table 1:**
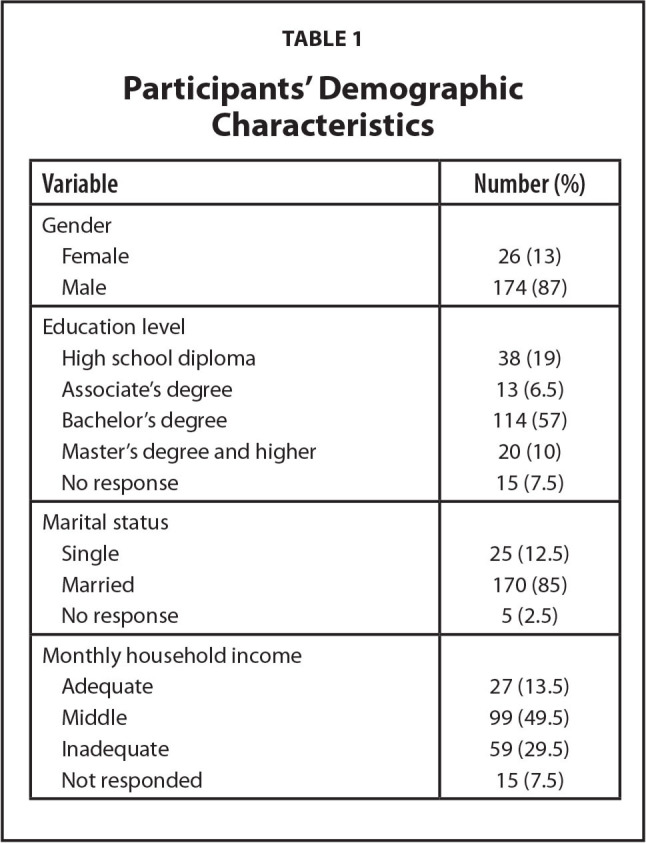
Participants' Demographic Characteristics

**Variable**	**Number (%)**

Gender	
Female	26 (13)
Male	174 (87)

Education level	
High school diploma	38 (19)
Associate's degree	13 (6.5)
Bachelor's degree	114 (57)
Master's degree and higher	20 (10)
No response	15 (7.5)

Marital status	
Single	25 (12.5)
Married	170 (85)
No response	5 (2.5)

Monthly household income	
Adequate	27 (13.5)
Middle	99 (49.5)
Inadequate	59 (29.5)
Not responded	15 (7.5)

### Construct Validity: Confirmatory Factor Analysis

**Figure 1.**
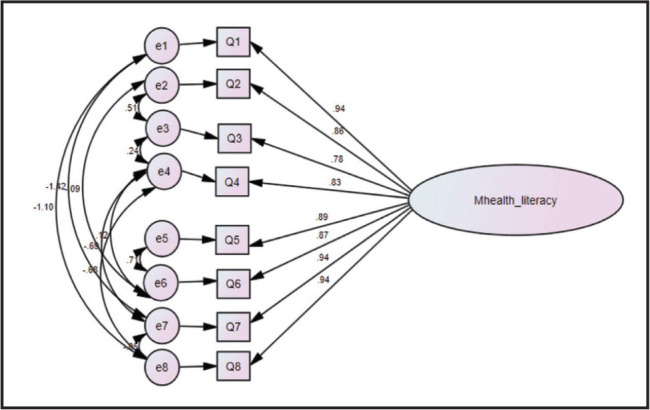
Confirmatory factor analysis of one-factor model of mHEALS.

**Figure 2.**
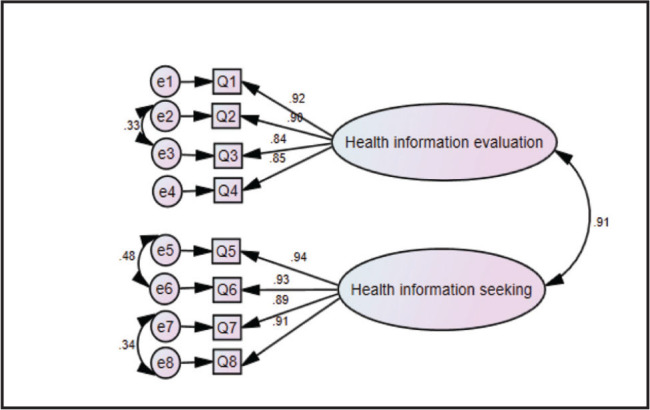
Confirmatory factor analysis of two-factor model of mHEALS.

A CFA was conducted to test the one-factor model structure (**Figure 1**) and then compared with the two-factor model structure (**Figure 2**). The result of the CFA showed that the one-factor model had a better fit to the data than the two-factor model (G*FI* = .985, C*FI* = .999, N*FI* = .994, T*LI* = .996, and RMS*EA* = .038) (**Table 2**).

**Table 2:**
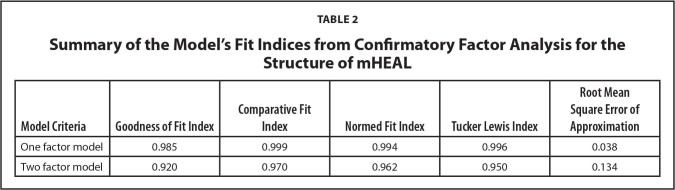
Summary of the Model's Fit Indices from Confirmatory Factor Analysis for the Structure of mHEAL

**Model Criteria**	**Goodness of Fit Index**	**Comparative Fit Index**	**Normed Fit Index**	**Tucker Lewis Index**	**Root Mean Square Error of Approximation**
One factor model	0.985	0.999	0.994	0.996	0.038
Two factor model	0.920	0.970	0.962	0.950	0.134

### Reliability

Cronbach's alpha test was used to determine the scale's internal consistency. Cronbach's alpha values for the first factor health information seeking and the second factor health information evaluation were obtained at 0.962 and 0.935, respectively, and for the total scale at 0.964.

## Discussion

The present study evaluated the psychometric properties of the Persian version of mHEALS among the workers of an automotive metal sheet factory in Iran. The Persian version of mHEALS had acceptable psychometric indices and no item was removed from the scale at different psychometric stages. In this study, the mHEALS shows good internal consistency and reliability. CFA yielded a one-factor model that provided a better fit to the data than the two-factor model. In a cross-sectional study, Bazm et al. (2016) examined the validity and reliability of the Iranian version of the e-Health Literacy Scale. In their study, Cronbach's alpha coefficient was calculated at 0.88 (Bazm et al., 2016). Moreover, eHealth literacy tools in other languages have been reported to have high reliability, with Cronbach's alpha coefficients higher than 0.88 (Diviani et al., 2017; Chang & Schulz, 2018). In the study of Wångdahl et al. (2020), the Swedish eHealth literacy scale consisted of 8 items that had acceptable construct validity.

Some similar studies have reported a one-factor (Chang & Schulz, 2018, Ma & Wu, 2019) or three-factor (Paige et al., 2017; Sudbury-Riley et al., 2017;) construct for eHealth literacy. It has been stated that tool translation may change the main meaning that can affect the concepts conceived by the respondents (Paige et al., 2017). In addition, eHealth tools were first designed before the advent of social media, which completely changed people's interaction with health information, which may affect the construct of eHealth literacy (Ziegel, 2003). The RMSEA value supports the modified two-factor model with better administration results than other models.

Chang and Schultz (2018) reported that deletion of items 7 or 8 could improve the reliability of Chinese eHealth Literacy Scale, which has not been reported in other studies. In the study of Diviani et al. (2017), respondents rated item 4 as the easiest and item 8 as the most difficult. For the first time in Iran, this study examined the psychometric properties of the Persian version of the mHEALS in workers, which showed acceptable reliability and validity for the tool.

One of the limitations of this study was the completion of the questionnaire by a limited number of women and fairly highly educated workers, which affects the results' generaliz-ability to the majority of society. In addition, only one mode of administration (self-report) was implemented that may be affected by the recall bias. This tool can be used to assess the health literacy status of Iranian workers and thus identify barriers to promoting their mHealth literacy and planning for and developing interventions to improve them.

## Conclusion

To the best of our knowledge, this is the first study that examined the psychometric properties of the mHEALS among an automotive metal sheet factory's worker in Iran. The 8-items one-factor mHEALS had satisfactory construct validity and internal consistency for our participants. As the tool measured mHealth literacy satisfactorily in our study, it is recommended to use it in different demographic groups to evaluate its generalizability so that its predictive validity may be ascertained in various settings.
